# Prospective Study in a Porcine Model of *Sarcoptes scabiei* Indicates the Association of Th2 and Th17 Pathways with the Clinical Severity of Scabies

**DOI:** 10.1371/journal.pntd.0003498

**Published:** 2015-03-02

**Authors:** Kate E. Mounsey, Hugh C. Murray, Helle Bielefeldt-Ohmann, Cielo Pasay, Deborah C. Holt, Bart J. Currie, Shelley F. Walton, James S. McCarthy

**Affiliations:** 1 Inflammation & Healing Research Cluster, School of Health and Sport Sciences, University of the Sunshine Coast, Maroochydore, Queensland, Australia; 2 Infectious Diseases Division, QIMR Berghofer Medical Research Institute, Herston, Queensland, Australia; 3 School of Veterinary Sciences, University of Queensland, Gatton, Queensland, Australia; 4 Global and Tropical Health Division, Menzies School of Health Research, Charles Darwin University, Darwin, Northern Territory, Australia; 5 School of Medicine, University of Queensland, Herston, Queensland, Australia; University of California San Diego School of Medicine, UNITED STATES

## Abstract

**Background:**

Understanding of scabies immunopathology has been hampered by the inability to undertake longitudinal studies in humans. Pigs are a useful animal model for scabies, and show clinical and immunologic changes similar to those in humans. Crusted scabies can be readily established in pigs by treatment with the glucocorticoid dexamethasone (Dex).

**Methodology/ Principal Findings:**

Prospective study of 24 pigs in four groups: a) Scabies+/Dex+, b) Scabies+/Dex-, c) Scabies-/Dex+ and d) Scabies-/Dex-. Clinical symptoms were monitored. Histological profiling and transcriptional analysis of skin biopsies was undertaken to compare changes in cell infiltrates and representative cytokines. A range of clinical responses to *Sarcoptes scabiei* were observed in Dex treated and non-immunosuppressed pigs. An association was confirmed between disease severity and transcription of the Th2 cytokines IL-4 and IL-13, and up-regulation of the Th17 cytokines IL-17 and IL-23 in pigs with crusted scabies. Immunohistochemistry revealed marked infiltration of lymphocytes and mast cells, and strong staining for IL-17.

**Conclusions/ Significance:**

While an allergic Th2 type response to scabies has been previously described, these results suggest that IL-17 related pathways may also contribute to immunopathology of crusted scabies. This may lead to new strategies to protect vulnerable subjects from contracting recurrent crusted scabies.

## Author Summary

Scabies is a neglected tropical skin disease caused by the tiny parasitic mite *Sarcoptes scabiei*. Scabies is common in developing countries, and scabies outbreaks also occur in institutional settings worldwide. Scabies often underlies secondary bacterial skin infection and resulting complications, and is thus associated with considerable morbidity. Crusted scabies is a an extremely severe and debilitating clinical form of the disease, but host immune responses leading to the development of crusted or ordinary scabies are poorly understood. This is largely due to limited access to clinical samples, and the difficulty in monitoring the progression of infestation in human patients. We have overcome this challenge by using a pig model of scabies infestation, since pigs and humans with scabies display clinical and immunological similarities. In this study, we undertook longitudinal analysis of clinical, histological and molecular immunological changes in pigs experimentally infected with scabies. We confirmed that disease severity was associated with a pronounced allergic, Th2 immune response, as previously reported. In a novel finding, we showed that the Th17 associated cytokines interleukin-17 and interleukin-23 were also associated with the development of crusted scabies. This may lead to new immunotherapeutic strategies to protect vulnerable subjects from contracting recurrent crusted scabies.

## Introduction

*Sarcoptes scabiei* infestation is associated with considerable global morbidity [1]. The disease is prevalent in overcrowded living conditions, with the highest disease burdens seen in young children [2]. The link between scabies, secondary bacterial infection and sequleae such as post-streptococcal glomerulonephritis [3] has resulted in efforts to reduce the prevalence of scabies in endemic communities.

Ordinary scabies manifests as a localised or general rash with low mite burden (<20 mites). Crusted (Norwegian) scabies is a less common but debilitating form, with proliferation of mites, hyperkeratosis, and risk of serious secondary infection. Crusted scabies requires aggressive treatment, and recrudescence and reinfestation are common [4].

Factors underlying the development of crusted scabies include iatrogenic immunosuppression and other immunosuppressive conditions such as HIV, HTLV-I and systemic lupus erythematosus [5–7]. The disease has also been described in those with no immune deficit [7–9], and reasons for crusted scabies development in this cohort are unknown. Limited humoral and cellular studies conducted to date suggest that crusted scabies is associated with a non-protective allergic T helper (Th) 2 response [10–12], but these are confounded by difficulties in assessing clinical severity [13], and the fact that patients present at an advanced stage of infestation.

Prospective studies are necessary to gain meaningful insights into immune responses driving crusted scabies. Scabies is associated with delayed onset of symptoms (4–6 weeks) in primary infestation, and several studies show that *S*. *scabiei* is capable of down regulating cytokine expression, likely suppressing early immune responses to allow mites to establish [14–18]. However, these studies were mostly *in-vitro*, utilising mite extracts and cultured cells or skin equivalents. We have recently developed a porcine model to investigate aspects of scabies immunology [19–21]. Pigs are a natural host of *S*. *scabiei* var *suis*, developing similar clinical manifestations to humans, including crusted and ordinary scabies. In this study we conducted transcriptional analysis of representative Th1, Th2, and Th17 pathway cytokines in the skin of infected pigs at several time points post infestation, and assessed skin biopsies with different clinical phenotypes by immunohistochemistry for inflammatory markers.

## Methods

### Porcine trial

Animal ethics approval was obtained from the QIMR Berghofer Medical Research Institute (Approval 1266) and the Queensland Department of Agriculture, Forestry and Fisheries (Approval SA 2009/07/294). Animals were handled in accordance with good animal practice as defined by the Australian code of practice for the care and use of animals for scientific purposes and the Australian National Health and Medical Research Council’s Animal Code of Practice.

Details regarding trial design have been described elsewhere [20]. The study involved 24 female piglets in four treatment groups (n = 6 per group). Group A: treated daily with 0.25mg/kg oral Dexamethasone (Dex) and ears infested with approximately 2,000 *S*. *scabiei* var *suis* mites. Group B: infested with approximately 2,000 mites. Group C: treated daily with 0.25mg/kg Dex (Dex only control). Group D: No Dex or mite infestation (negative control). While the infested and non-infested groups were kept isolated from each other, the allocation of individual pigs to pens was random, meaning that Dex and non-Dex pigs were housed together. Skin lesions were scored weekly on a 1–8 scale (1 = minimal change, >4 = development of crusts, 8 = extensive crusting. Skin scrapings were collected from a 2cm^2^ ear region of each pig fortnightly to approximate mite burden, as described previously [19]. Mite burden was graded as follows: – = no mites, + <20 mites/scrape, ++ = >20–100 mites/scrape, +++ = >100 mites/scrape. Two adjacent 3mm skin punch biopsies were collected from the ears of all pigs at week 0, 4, 8, and 12 post-infestation. At this size biopsies healed rapidly with minimal scarring. For infested pigs, biopsies were taken directly from lesional areas where scabies infestation was apparent. Biopsies were full skin thickness, including hyperkeratotic areas (if apparent), epidermis, dermis, and underlying ear cartilage. One biopsy was stored in RNA Later reagent (Life Technologies) and kept at-80°C. The second was collected into 10% neutral buffered formalin, fixed for 24 hours, transferred to 70% ethanol, and kept at 4°C.

### Histological and immunohistochemical examination of skin biopsies

Ten pigs were selected for analysis- two from groups A, C and D, and four from Group B. These represented different clinical phenotypes—crusted scabies, ordinary scabies, and non-infested, based on clinical presentation and mite burden. The four pigs in Group B included two pigs with crusted scabies (designated Group B+ in subsequent results). Serial sections (4–7μM) were cut from paraffin embedded biopsies, dewaxed and stained with hemotoxylin and eosin. Slides were examined for cellular, structural and vascular changes (Table 1) and each parameter allocated a score of 0–5 where 0 = minimal change and 5 = extensive change. The inspecting pathologist was blinded to the allocated group.

**Table 1:** Histopathological changes measured in this study.

Epidermis
Basal cell hyperplasia/acanthosis^a^
Rete peg hypertrophy
Apoptosis/necrosis/erosion
Microabscesses^b^
Subepidermal clefts^c^
Ortho-hyperkeratosis^d^
Para-hyperkeratosis^e^
Transudation
Ulceration
**/45** ^**f**^
**Dermis**
Edema
Collagen degeneration
Neovascularization
Vasculitis/transendothelial migration
Granulocyte infiltration
Mononuclear cell infiltration
Mast cell degranulation
**/35**
**TOTAL PATHOLOGY SCORE /80**

^a^ Acanthosis/basal cell hyperplasia: thickening of the prickle layer or increase in basal cell number;

^b^ Microabcess: very small circumscribed collection of white blood cells;

^c^ Subepidermal clefting: localised or generalised detachment of the epidermis.

^d^ Orthohyperkeratosis: thickening of the outermost layer of the epidermis, cells are anucleated;

^e^ Para-hyperkeratosis: thickening of the outermost layer of the cells, cells are nucleated.

^f^ Each parameter was scored from 0–5, where 0 = within normal limits, 1 = minimal change, 2 = mild change, 3 = moderate change, 4 = severe focal change, 5 = severe extensive change

Immunohistochemistry was undertaken to characterise the cellular infiltrate, as well as for IL17 cytokine staining. T cell numbers were assessed by staining with anti-CD3 antibody. Dewaxed sections were incubated with high pH antigen retrieval solution (Dako pH 9.0) and blocked with purified casein (Medical background sniper, Biocare). Rabbit anti-human CD3 antibody (Biocare), previously established to cross-react with pig tissue, was diluted 1:275 and incubated overnight at room temperature. Sections were washed 3 times for 5 minutes in phosphate buffered saline. Anti-rabbit HRP secondary antibody (Vector labs) was applied for 30 minutes, washed as above, and the HRP substrate Novared (Vector labs) applied and developed for 5 minutes. For mast cell visualisation, sections were stained with toluidine blue. Analysis was performed on CD3 and toluidine blue stained sections by scanning slides with an Aperio XT scanner. Positively labelled cells were counted in 10 fields at 20 X magnification. Cell concentration (cell/mm^2^) was calculated for each field by dividing the count total by the area of the field (0.234mm^2^) and the value for each of the 10 fields averaged.

For IL-17 detection, dewaxed sections were blocked for endogenous peroxidases with 1.0% H_2_O_2_, 0.1% sodium azide for 10 minutes. Sections were incubated in citrate pH 6.0 antigen retrieval buffer at 97°C, and blocked in 4% skim milk powder, followed by purified casein (Medical background sniper, Biocare) plus 10% normal goat serum and 1.0% BSA. Polyclonal rabbit anti-human IL-17 (Abcam, 1mg/mL), diluted 1:100, was applied for 2 hours at room temperature. This antibody was derived from a synthetic 19 amino acid peptide of human IL-17A, and based on sequence conservation was predicted to be cross reactive with its pig homologue. Sections were washed 3 times for 5 minutes in tris buffered saline. Anti-rabbit-HRP secondary antibody (Mach2, Biocare) was applied for 45 minutes, sections washed as above and developed with 3,3'-diaminobenzidine (DAB) with H_2_O_2_ as substrate for 5 to 10 minutes. Staining intensity was assessed qualitatively on a scale of 0–4 by a dermatologist blinded to the allocated group.

### RNA extraction and reverse transcription

Biopsies stored in RNA Later were thawed and homogenised in 600μL TRIzol reagent (Life Technologies) using the Tissue Lyser II homogeniser (Qiagen). Phase separation with TRIzol was undertaken according to the manufacturers’ protocol. The aqueous phase was column purified as per the manufacturers’ protocol (PureLink RNA mini-kit, Life Technologies), including DNAse digestion. RNA was eluted in RNAse free dH_2_0 and stored at-80°C. RNA quantity and integrity was assessed using the Nanodrop ND2000 spectrophotometer (Nanodrop Technologies) and Agilent Bioanalyzer RNA 6000 nano-kit (Agilent Technologies). One μg of purified total RNA was reverse transcribed to cDNA using the QuantiTect reverse transcription kit (Qiagen). The cDNA was diluted 1:4 in dH_2_0 and stored at-20°C.

### Quantitative PCR

Primers were designed using Primer3 software (http://frodo.wi.mit.edu/) (Table 2). Hypoxanthine phosphoribosyl transferase 1 (HPRT1) was selected as a reference gene, as this gene is proposed to be stable under different environmental conditions [22]. Gradient PCR to test optimal annealing temperatures was performed using control skin cDNA. PCR products were purified (Roche), cloned (pGEM-T, Promega) and sequenced (Big Dye 3.1, Applied Biosystems). Sequence identity was confirmed using BLASTx (http://blast.ncbi.nlm.nih.gov/).

**Table 2:** Primer sequences and amplicon details for porcine qPCR.

Gene	Primer sequence (5’-3’)	Amplicon size (bp)	Genbank accession
HPRT1	F: GCAGCCCCAGCGTCGTGATT	142bp	NM_001032376.2
	R: CGAGCAAGCCGTTCAGTCCTGT		
IFNγ	F: CCAGGCCATTCAAAGGAGCATGGA	140bp	NM_213948.1
	R: GGCTTTGCGCTGGATCTGCAGA		
TGF-β	F: CACGGCATGAACCGGCCCTT	148bp	NM_214015.1
	R: TGTAGAGCTGCCGCACGCAG		
IL-2	F: ACTGGAGCCATTGCTGCTGGA	117bp	NM_213861.1
	R: TCTGTAGCCTGCTTGGGCATGTA		
IL-4	F: AGAACTCGTGCATGGAGCTGCC	100bp	NM_214123.1
	R: TGCCGAAGCACAGTCGAGGC		
IL-5	F: TGGAGCTGCCTACGTTAGTGCCA	109bp	NM_214205.1
	R: CCCATCGCCTATCAGCAGAGTTCG		
IL-6	F: ACCCCACCACAAATGCCGGC	123bp	NM_214399.1
	R: TGGCCCTCAGGCTGAACTGC		
IL-10	F: CTGGAAGACGTAATGCCGAAG	121bp	NM_214041.1
	R: GCAGAAATTGATGACAGCGC		
IL-13	F: ACCTGCTTTGGTGGCCTCGC	135bp	NM_213803.1
	R: GCTCCACACCATGCTGCCGT		
IL-17	F: CGGAGCACACCTGCCAGACG	121bp	NM_001005729.1
	R: GGCTGCACTTGGCCTCCCAG		
IL-23	F: ACAGCAGCTCTGCACGCTGG	125bp	NM_001130236.1
	R: CACAGCCATCCCCGCACTGG		

To assess amplification efficiency, plasmids containing the gene of interest were linearised and serially diluted. qPCR was done using the QuantiTect SYBR green PCR kit (Qiagen). Reactions contained 1 X SYBR green master mix, 0.4μM primers, 1μL diluted plasmid DNA and dH_2_0 to total volume of 10μL. Reactions were cycled in the Rotor Gene 6000 real-time cycler (Qiagen). Cycling conditions were: initial denaturation 95°C, 15 min, followed by 40 cycles of 94°C, 15 s; 56°C, 30 s; 72°C, 30 s; with data acquisition at 76°C, 20 s. Standard curves, melting temperature and efficiency calculations were produced using the Rotor Gene software.

qPCR was run on the cDNA samples for the gene of interest in parallel with HPRT1, allowing for normalisation. A no-RT control containing RNA as template was used to confirm that co-amplification of genomic DNA was not occurring. Each PCR also included a no template control. Reactions were performed in duplicate. Individual reaction mixtures were as above, except that 2μL cDNA was used as template.

To measure transcriptional differences between treatment groups relative to the untreated, uninfested control group (Group D), the ΔΔCt formula was used, corrected for PCR efficiency [23]. Significance of differences between groups was assessed using unpaired T tests at each time-point using GraphPad Prism version 5.0 (GraphPad Software, Inc.).

## Results

### Clinical development

**Fig 1:**
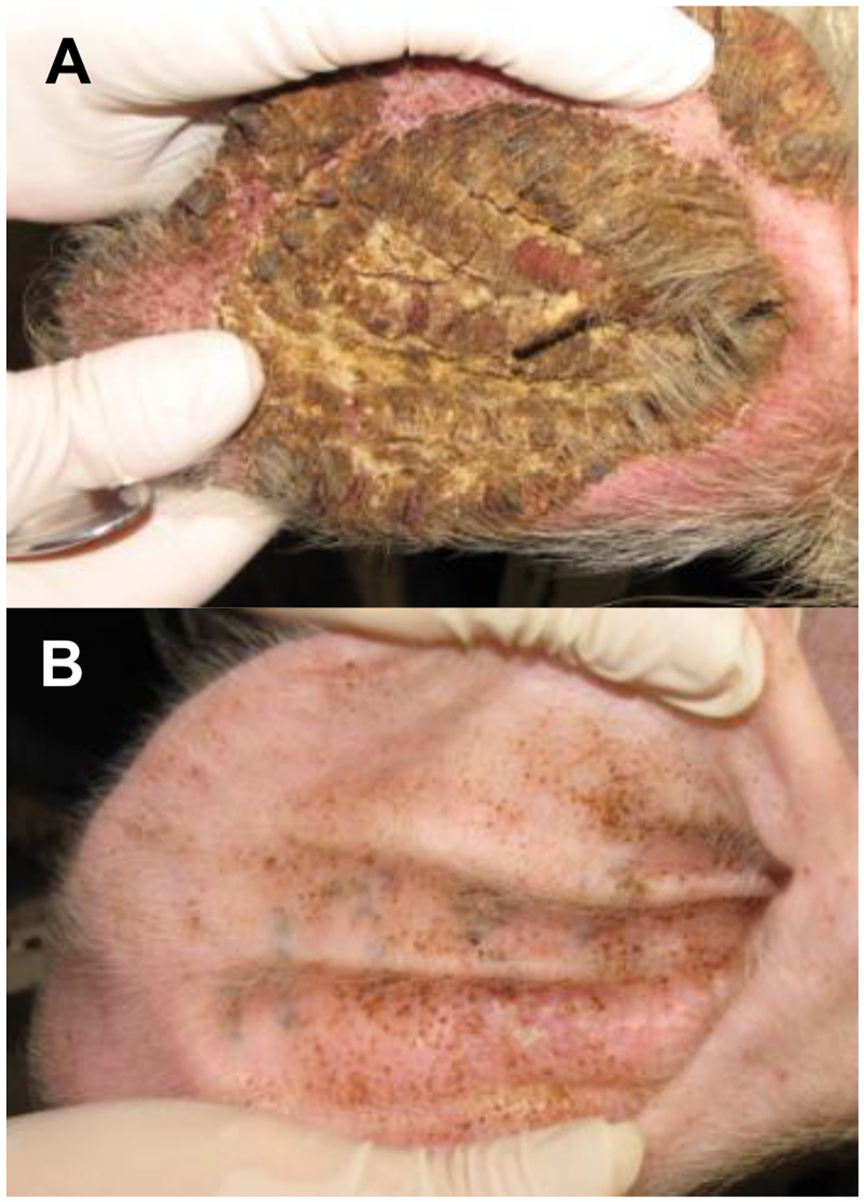
Clinical appearance of crusted (A) & ordinary scabies (B) at week 12 post infestation with *Sarcoptes scabiei*.

Details of the clinical phenotypes observed in the trial are presented elsewhere [20]. All pigs in Group A developed crusted mange (skin score >4) from weeks 8–24 post-infestation (Fig 1A). Two pigs in group B also developed crusted mange in the absence of Dex immunosuppression (designated as group B+ in subsequent results). The remaining pigs in group B developed an acute reaction, with lesion severity 1–4, peaking at weeks 8–12 before declining (Fig 1B). Mite counts were associated with lesion scores, with positive scrapings obtained from 4/6 pigs in group A, and 3/6 pigs in group B in week 4. From week 8, differences between the groups became more apparent, with most pigs in group A having heavy mite infestations. In group B, 2 pigs developed heavy infestations (Group B+) while 4 pigs had low-moderate infestations. Pigs in the non-infested groups did not develop skin lesions nor have detectable mites at any time (Table 3).

**Table 3:** Approximate *Sarcoptes scabiei* numbers in skin scrapings collected from trial pigs.^a^

	Group A				Group B^b^				Group C				Group D			
Week	0	4	8	12	0	4	8	12	0	4	8	12	0	4	8	12
Pig 1^b^	-	+	+++	+++	-	+	+++	+++	-	-	-	-	-	-	-	-
Pig 2^b^	-	+	+++	+++	-	-	+++	+++	-	-	-	-	-	-	-	-
Pig 3	-	+	+++	+++	-	-	-	++	-	-	-	-	-	-	-	-
Pig 4	-	-	++	+++	-	-	-	+	-	-	-	-	-	-	-	-
Pig 5	-	-	+++	+++	-	+	++	+	-	-	-	-	-	-	-	-
Pig 6	-	+++	+++	+++	-	+	+	+	-	-	-	-	-	-	-	-

^a^ Mite counts of a 2cm^2^ skin scraping.- = no mites + = <20 mites ++ = 20–100 mites +++ = >100 mites,

^b^ Pigs 1 & 2 in Group B are referred to as Group B+ in text and figures

### Histological changes in representative pigs

**Fig 2:**
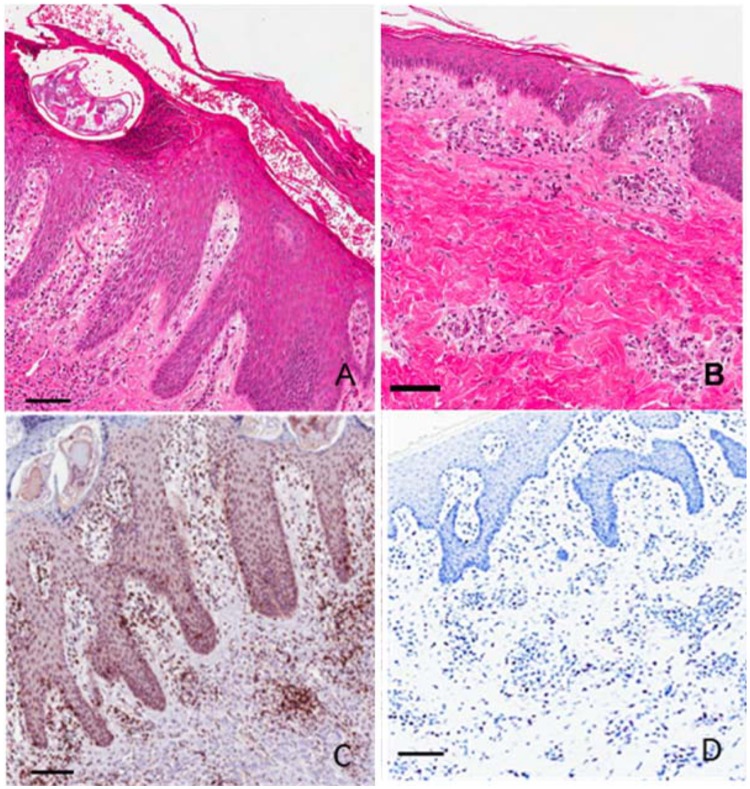
Representative histology and immunohistochemistry of skin lesions at week 12 post infestation with *Sarcoptes scabiei*. (A) Crusted scabies, hematoxylin and eosin stain; (B) Ordinary scabies, hematoxylin and eosin stain; (C) Crusted scabies, anti-CD3+ antibody T cell stain; (D) Crusted scabies, toluidine blue mast cell stain. Scale bars = 100μM.

**General histopathology**. Major epidermal changes characteristic of severe crusted *S*. *scabiei* infestation included acanthosis, rete peg hypertrophy and para-hyperkeratosis (Fig 2A). Other changes included apoptosis / necrosis /erosion, microabscesses and transudation (Table 4). At the dermal level pathology included edema, vasculitis, and infiltrates of granulocytes and monocytes. The level of pathology was associated with clinical severity, with the greatest changes observed in Groups A & B+. Group A had fewer histological changes at 4 weeks, but more dramatic change at 8 and 12 weeks. Histological changes in group B pigs that clinically had self-limiting infestation peaked at week 4 and were reduced at week 8 and 12. Minor changes were observed in one pig in group C (thickening, ortho-hyperkeratosis, minor mononuclear infiltrate). No histological changes were apparent in the group D, Table 4).

**Table 4:** Summary scores of histological changes in representative mange infested pigs.^a^

	Group A				Group B^b^				Group C				Group D			
Week	0	4	8	12	0	4	8	12	0	4	8	12	0	4	8	12
**Epidermal changes (/45)**																
Pig 1^b^	0	0	16	19	0	14	14	3	0	0	0	6	0	0	0	0
Pig 2^b^	0	0	16	20	0	0	18	13	0	0	0	0	0	0	0	0
Pig 3					0	8	4	0								
Pig 4					0	0	0	7								
**Dermal changes (/35)**																
Pig 1^b^	0	2	8	10	0	20	11	7	0	0	0	1	0	0	0	0
Pig 2^b^	0	3	19	21	0	0	17	16	0	0	0	0	0	0	0	0
Pig 3					0	10	6	1								
Pig 4					0	0	0	6								
**Interleukin-17 staining intensity** ^**c**^																
Pig 1^b^	-	+	++	+++	-	-	++	+	+	+	-	-	-	-	-	+
Pig 2^b^	-	+	+++	++++	-	-	++	++	+	+	-	-	-	-	-	-
Pig 3					+	-	-	+								
Pig 4					-	+	+	++								

^a^ Individual parameters assessed listed in table 1,

^b^ Pigs 1 & 2 in Group B are referred to as Group B+ in text,

^c^ Assessed qualitatively, - = no staining, ++++ = very strong staining

**Fig 3:**
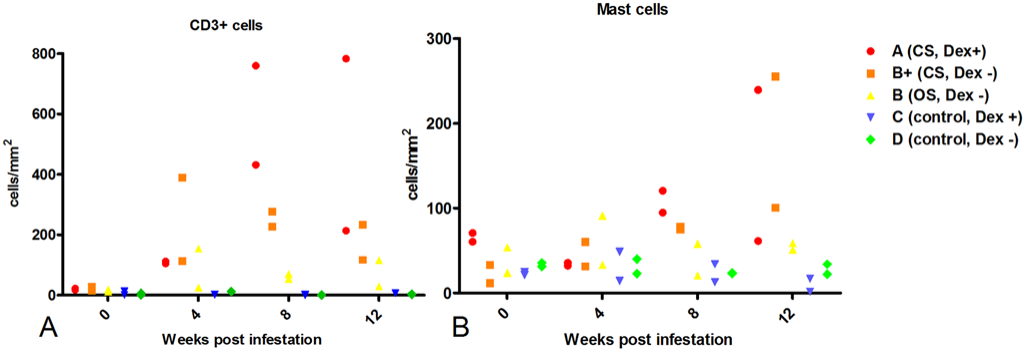
Numbers of CD3+ cells (A) and mast cells (B) in skin biopsies collected from representative pigs at week 0, 4, 8 and 12 post-infestation with *Sarcoptes scabiei*. Group A n = 2, B+ n = 2, B n = 2, C n = 2, D n = 2. Group B+ = pigs in group B that developed crusted scabies based on mite counts and lesion scores.

**CD3 immunolabeling**. Pigs in Groups A & B had increased T cell infiltrates relative to non-infested pigs in groups C & D as ascertained by CD3 immunolabeling (Fig 2C, 3A). Positive cells aggregated in a perivascular pattern in the papillary and reticular dermis and in the stratum basale and stratum spinosum of the epidermis. This increase was most marked in pigs in Group A and B+. Maximal infiltration was observed in pigs in group A at weeks 8 and 12, in Group B+ at weeks 4 and 8, and in Group B at week 4 (Fig 3A).

**Mast cell staining**. Mast cell numbers in crusted pigs were increased relative to non-infested controls at weeks 8 and 12 (Fig 2D, 3B). Positively stained cells were perivascular in the papillary and reticular dermis. No stained cells were present in the epidermis. Mast cell numbers in pigs with ordinary scabies did not change dramatically over the course of infestation, but were slightly elevated relative to other groups at week 4.

**Fig 4:**
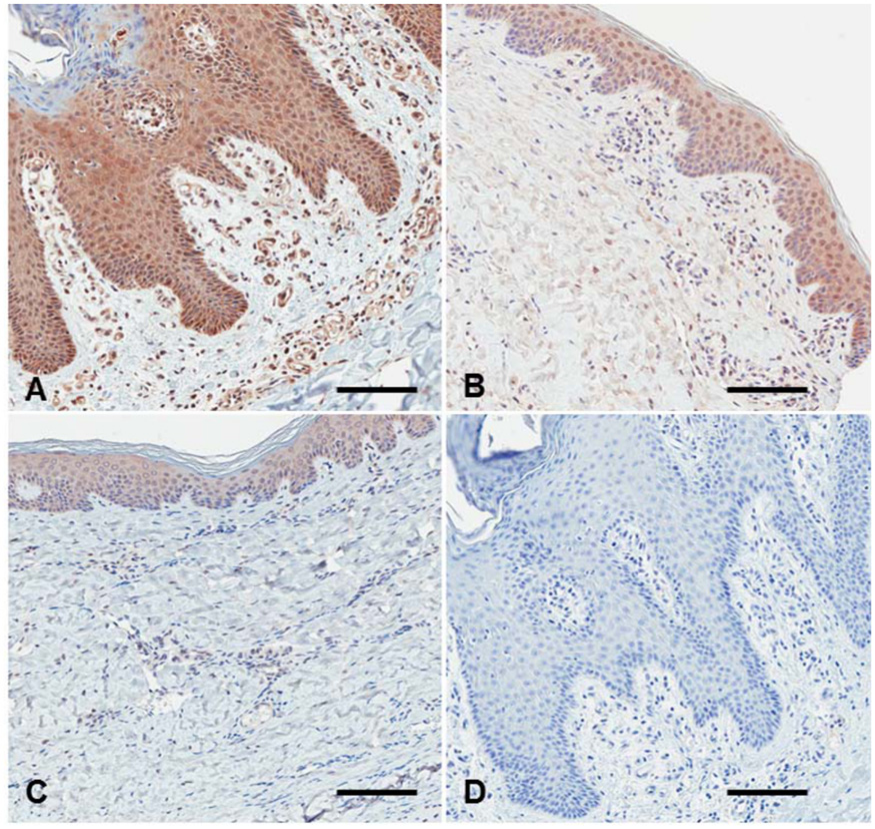
Representative IL-17 staining of skin lesion at week 12 post infestation with *Sarcoptes scabiei*. (A) Crusted scabies, (B) Ordinary scabies, (C) Non-infested, (D) anti-interleukin-17 isotype control antibody. Scale bars = 100μM.

**IL-17 immunolabeling**. IL-17 staining l was moderate to intense in pigs with crusted scabies at weeks 8 and 12 (Group A, B+), while low to moderate in pigs with ordinary scabies (Group B) and minimal in non-infested pigs (Table 4, Fig 4). Where positive, IL-17 labeling was widespread and generally dispersed and located in dermal cells, stratum basale and stratum spinosum, as well as within vessels. There was also a strong signal in keratinocytes (Fig 4). No signal was observed with the isotype control antibody (Fig 4D).

### Changes in cytokine transcription

**Fig 5:**
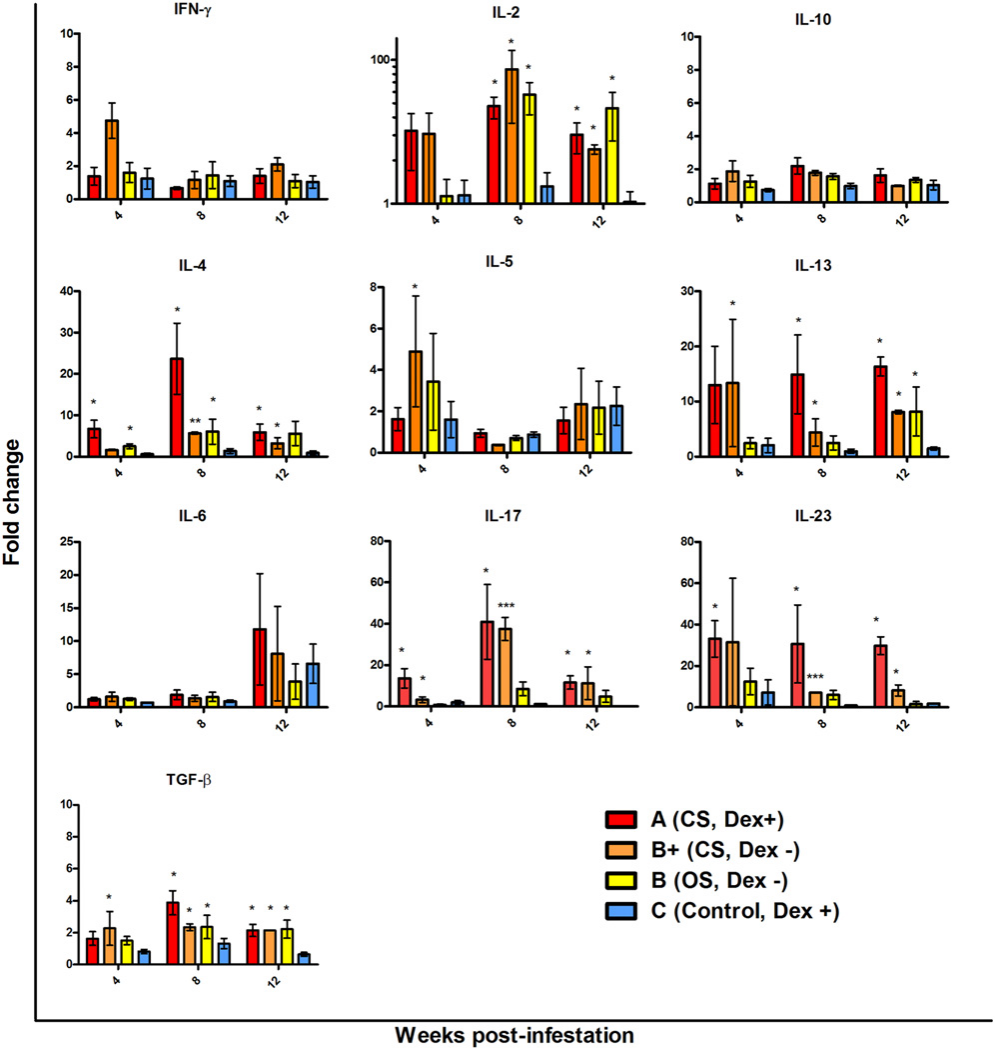
Changes in cytokine transcription in skin biopsies collected from all pigs at weeks 4, 8 and 12 post-infestation with *Sarcoptes scabiei*. Group A n = 6, B+ n = 2, B n = 4, C n = 6. qRT-PCR was performed on 1μg total RNA and transcription normalised to the housekeeping gene HPRT1. Relative fold-changes in gene transcription in treatment groups were calculated by comparison to control pigs in Group D (n = 6). * = p<0.05, ** = p<0.001, ***p<0.0001 vs control, unpaired T test. Bars represent mean +/- SEM. Abbreviations: IL, interleukin, IFN, interferon, TGF, transforming growth factor.

Scabies was associated with significant changes to several cytokines measured, including transforming growth factor β (TGF-β), interleukin (IL)-2, IL-4, IL-13, IL-17 and IL-23 (Fig 5). No significant changes to interferon γ (IFNγ), IL-5, IL-6 or IL-10 were detected. In the following section, while fold changes in transcription are noted, the Group B+ P-values are not reported due to the low numbers of pigs in this group limiting meaningful statistical interpretation. When comparing pigs with crusted scabies (Groups A and B+) to those with ordinary scabies (Group B) an increased magnitude of IL-13, IL-17 and IL-23 responses was observed from 4 weeks. IL-13 was increased both in Group A and B+ by 13-fold at 4 weeks (Group A p = 0.05), in Group A (15-fold, p = 0.002) and B+ (4-fold) at 8 weeks, and in Group A (16-fold, p = 0.009) and B+ (8-fold) at 12 weeks. By contrast in Group B pigs with ordinary scabies elevation of IL-13 was only observed at week 12 (8-fold, p = 0.03). We saw upregulation in the Th17 cytokines, IL-17 and IL-23 only in pigs with crusted scabies. IL-17 was significantly upregulated at all time points, most strongly at week 8 (Group A 41-fold, p = 0.009, Group B+ 37-fold). Upregulation of IL-23 was observed at all time points, with a 30-fold increase observed in Group A pigs from week 4 (p = 0.03).

Transcription of IL-4 was increased in all infected pigs at all time points, with the exception of group B+ at 4 weeks. The greatest elevation of IL-4 was in Group A pigs at 8 weeks (24-fold, p = 0.002). IL-2 levels also increased in all infected pigs from week 4, but the change only became significant after week 8. Similarlary TGB-β was modestly but signficantly upregulated in infected pigs at all time points, with the exception of Group A at week 4.

There were no significant differences between non-infected Dex +ve and Dex—ve pigs (Groups C & D), suggesting that the Dex had little impact on baseline levels of these cytokines in the skin.

## Discussion

Comparison of immune responses in scabies been confounded by the limited availability of clinical samples and standardisation problems related to differences in disease presentation and the existence of co-morbidities [24]. Animal models offer the ability to correlate clinical phenotype with immune parameters and to report the temporal development of immune responses. We observed that phenotypic differences between crusted and ordinary scabies in a porcine model were associated with differences in both the timing and magnitude of cytokine responses and histological changes.

Scabies became clinically apparent in infested pigs from week 4, with mite numbers correlated with the appearance of clinical lesions. As skin scrapings can have poor diagnostic sensitivity due to low mite numbers, and are difficult to perform in large herds or community studies, clinical appearance is more useful as a proxy measure of infestation level [25]. For example, while several pigs in Group B had negative skin scrapings at weeks 4 and 8, clinical scores indicated they were still infested. While most infested pigs had similar mite counts at week 4, pigs that developed crusted scabies had substantially increased mite numbers from week 8, while those with ordinary scabies maintained low or moderate numbers of mites. Notably, two pigs from group B developed crusted scabies in the absence of immunosuppression. While acknowledging the small number and consequent limited interpretation of results for Group B+, we elected to compare these pigs as a separate “subgroup”, as the development of crusted scabies in the absence of Dex immunosuppression is of interest. These clinical observations reflect what is well documented in the literature- while the majority of pigs with sarcoptic mange develop an ‘acute’ manifestation with clinical peak of around 8 weeks before a decline in skin lesions and mite numbers, indicative of a self limiting infestation, some pigs develop chronic hyperkeratotic mange akin to crusted scabies in humans. This reinforces the value of the porcine model to explore protective versus pathologic immune responses in scabies, and further studies by our group have focused on the further study of different clinical phenotypes in pigs not receiving Dex treatment [21].

As histological analysis of scabies lesions has been reported in the literature previously for both pigs and humans, we did not intend to undertake comprehensive histological comparisons in this study, but rather obtain a representative “snapshot” to link our clinical and molecular observations in different clinical phenotypes of scabies. Being mindful of the limited numbers of pigs examined, histopathology generally mirrored clinical observations. An exception was that pigs in Group A had delayed inflammatory responses at week 4 relative to Group B. As the pigs in Group B+ with crusted scabies also had inflammatory changes at week 4, these differences may be more attributable to Dex supressing early inflammatory responses rather than differences between crusted and ordinary scabies. From week 8 pathologic changes between the clinical phenotypes were more apparent, which was also reflected in CD3+ T cell numbers. Increased T lymphocytes in scabies lesions have also been reported in humans [26] and other animals [26–29]. Ongoing work has shown that the CD3+ T cell infiltrate in pigs with crusted scabies is comprised largely of γδ T cells and CD8+ T cells [21]. Although γδ T cells have not yet been examined in human scabies, CD8+ tropism has been observed in crusted scabies [30], while increased CD4+ cell infiltrates were associated with protective immunity in canine mange [28].

Crusted scabies was associated with increased mast cell numbers, most notably at week 12 post infestation. Mast cell numbers remained steady throughout the study in pigs with ordinary scabies. The presence of mast cells is consistent with previous findings [27,29,31,32]. The presence of mast cells, often with accompanying eosinophilia, is reflective of the allergic and immediate hypersensitivity component of the scabies immune response, particularly upon secondary exposure [31]. The role of mast cells and related high IgE levels in protective versus pathologic responses to scabies is yet to be resolved [33].

As well as general T cell proliferation and inflammatory markers such as IL-2 and TGF-β, crusted scabies was associated with a pronounced Th2 response. This was most evident with IL-13, and to a lesser extent, IL-4, whereas IL-5 was not signifigantly elevated. These findings are in accordance with cross-sectional studies on human patients [10], where peripheral blood mononuclear cells (PBMCs) from crusted scabies patients secreted more IL-5 and IL-13, and reduced IFNγ in response to stimulation with *S*. *scabiei* antigens [10]. While we did not see any transcriptional changes in IFNγ in the present study, this may be related to the timing of infestation, local versus peripheral responses, or primary versus secondary infestation. For example Lalli et al [34] found that while primary exposure to *S*. *scabiei* in mice was associated with an IL-4 response, secondary exposure following immunization was IFNγ oriented. Other studies by our group have shown increased CD4+ IFNγ+ T cells at one week post infestation in PBMCs from mange infested pigs [21].

This is the first study to measure temporal changes in cytokine levels in scabies infested skin. In studies undertaken on clinical patients, little information was available regarding duration of current infection and a key question was if elevated Th2 responses precede, or are simply a consequence of, the extreme antigen burden in crusted scabies [35]. Our studies show that Th2 elevation, particulary of IL-13, occured prior to the development of high mite burdens and before major clinical or histological differences between groups became evident.

The observation of increased IL-17 in the skin by immunohistochemistry and qPCR supports our recent findings of increased CD3+ IL-17+ cells in crusted scabies as determined by intracellular cytokine staining [21]. In this study, increased IL-17 was observed at week 15 post-infestation. Here, we show that transcriptional increases of IL-17 begin from as early as week 4 post-infestation. Again, this was prior to the development of strong clinical or inflammatory changes in the skin, suggesting that the IL-17 increase is associated with a dysregulated response rather than just a consequence of a changed inflammatory skin milieu.

IL-17 is a proinflammatory cytokine implicated with a number of allergic and inflammatory diseases. Traditionally associated with CD4+ T cells (Th17), IL-17 is also secreted by other innate and adaptive immune cells in the skin, including CD8+ T cells, γδ T cells, and mast cells [36]. While γδ cells are likely a major source of IL-17 in crusted scabies [21], the contribution of CD8+ and mast cells to local IL-17 production is still to be investigated. Regardless of the cell type, it is accepted that functional maturation and IL-17 secretion is promoted by increases in IL-23, secreted by dendritic cells, macrophages and keratinocytes, in the presence of TGB-β and IL-6 [36]. These are all present in scabies infested skin, supporting an IL-17 environment. Importantly, IL-23 was only increased in crusted scabies, potentially promoting the subsequent high levels of IL-17. Using human skin equivalents, Morgan and colleagues [15] demonstrated that *S*. *scabiei* promotes up-regulation of IL-23 from 48 hours post infestation.

It is suggested that increases in IL-17 could be the result of a dysregulated regulatory T (Treg)/Th17 balance, or due to a deficit in IL-10 [21]. While mite extracts are capable of inducing IL-10 secretion in human PBMCs [37], reduced IL-10 was observed in PBMCs isolated from crusted scabies patients relative to ordinary scabies [10]. In the current study there were no observable differences in IL-10 between crusted and ordinary scabies. A limitation was that other markers of Treg function were not examined. A role for IL-10 regulation of IL-17 is supported by studies in leishmaniasis, where blockade of IL-10 resulted in increased IL-17 and exacerbation of skin pathology [38]. Increased IL-4 is also reported to suppress IL-10, exacerbating syptoms of Th2 mediated atopic dermatitis [39].

An important consideration is the potential impact of Dex on the immune parameters investigated. It is accepted that the effects of Dex are pleotropic, with dose, timing and experimental system appearing to play a role. These preliminary findings need to be supported by larger studies with non-immunosupressed pigs with crusted scabies. While the utilisation of Dex to induce the clinical phenotype of crusted scabies somewhat confounds interpretation of the immunologic parameters measured in this study, the data obtained is still informative. Firstly, comparing immune responses in the crusted scabies phenotype in the presence and absnece of immunosupression assists in refining a common immunopathology, regardless of causation. Secondly, crusted scabies in humans frequently arises from corticosteroid use, so an understanding of immune responses and potential implications for immunotherapy under these conditions are of interest. Thirdly, the effects of Dex on specific aspects of the immune system remain poorly defined in both humans and animals, so this study adds value at a general level. In our study, pigs were maintained on a relatively low dose of Dex (0.25mg/kg), with others reporting that porcine immunne funtion was resistant to higher doses (2mg/kg) [40]. Despite the low dose, there are several factors whereby Dex may be conducive to the development of crusted scabies. Dex may promote Th2 bias, with increased IL-4 and decreased IFNγ [41–43]. Other studies report Dex inhibition of Th2 responses [44,45] but again, these differences may be in part explained by the concentration used, with low doses stimulating, and high doses inhibiting IL-4 [46]. Of particular relevance, populations of double positive Th2/Th17 cells secreting IL-4 and IL-17 have been identified in severe asthma, and these cells were insensitive to Dex [47]. Dex treatment may decrease FoxP3+ CD4+ T cells [48], possibly causing further amplification of Th2 and Th17 pathways and promoting the development of crusted scabies.

This study contributes to the limited knowledge regarding the immunopathogenesis of crusted scabies, with a theme for involvement of Th2 and Th17 related cytokines now emerging, although numbers of pigs and human patients studied remains small. It is now important to gain more detailed insights into pathways of immune dysregulation in crusted scabies, particularly the contribution of regulatory T cells. Longitudinal studies are also needed earlier in infestation, prior to the development of clinical symptoms. Finally, studies where pigs are treated, then reinfested, would be of value to compare primary versus secondary immune responses to *S*. *scabiei*.
